# A Feasibility Study Investigating a Topical Preparation as Novel Adjunct Treatment for the Symptomatic Management of Vulvovaginal Skin Conditions

**DOI:** 10.1089/whr.2024.0026

**Published:** 2024-05-17

**Authors:** Philip Hall

**Affiliations:** The Pelvic Medicine Center, *St Andrew’s War Memorial Hospital*, Brisbane, Australia.

**Keywords:** lichen sclerosus, vulvovaginal atrophy, gel, dryness, dyspareunia

## Abstract

**Objective:**

The study aimed to investigate the feasibility of a newly available topical gel in improving the symptoms of various vulvovaginal skin conditions (NCT05396261).

**Methods:**

Fifty-two women with diagnosed lichen sclerosus, lichen simplex chronicus, or genitourinary syndrome of menopause participated in this prospective single-arm feasibility study. Consented patients applied the product daily internally and externally to the genital area for approximately 6 months (short-term) and optionally up to 2 years (long-term). Outcome measures included patient-rated symptoms, investigator-assessed clinical signs, and visual severity of pathology of these vulvovaginal conditions. Clinical outcomes, patient adherence to the treatment, and adverse events were assessed, and the statistical analysis was split according to short-term and long-term treatment.

**Results:**

The majority of patients enrolled in the study suffered from an uncontrolled disease (90.4%). All patients showed significant improvement in all patient-rated symptoms (*p* < 0.001), overall clinical signs (*p* < 0.001), and visual severity of pathology (*p* < 0.001) short-term. These favorable results were maintained from month 6 up to 2 years. Patient compliance was high, and no adverse events were reported with use of the investigational product.

**Conclusions:**

This topical medical device could be an effective symptomatic management option for women suffering from various vulvovaginal conditions.

## Introduction

The wellbeing and sexual function of postmenopausal women can be significantly affected by vulvovaginal symptoms, such as dryness, burning, itching, and dyspareunia, along with dysuria, urinary frequency, and recurrent infections.^1^ The most common genital disorders associated with these symptoms include lichenoid vulvovaginal dermatoses, such as lichen sclerosus (LS) and lichen simplex chronicus (LSC), as well as genitourinary syndrome of menopause (GSM).^2^ These disorders are often chronic in nature, reported to last over 3 years or longer for 55% of surveyed women, although their prevalence is likely underestimated.^3^ This is especially concerning, considering that 45%–60% of all women suffer from these debilitating symptoms at some point in their life.^3,4^ As the pattern of symptoms between these conditions are similar and overlapping disorders may occur, distinguishing between these conditions is often difficult.

Vulvovaginal conditions are mainly characterized by inflammation and typically lead to atrophy, fibrosis, and potential scarring. Clinically, LS is characterized by white sclerotic patches that later combine to form areas that are ivory white or shiny in color.^5^ Symptoms associated with LS include pain, pruritus, dyspareunia, constipation, painful urination, or defecation, as well as tissue changes that result in atrophy and distortion of anatomical structures.^5^ As opposed to other dermatoses, vulvar LS is associated with an increased risk of developing squamous cell carcinoma, especially in patients with poorly controlled disease.^6^

LSC is commonly present on hair-bearing surfaces. Pruritus is the main symptom, where chronic scratching injures the skin barrier, subsequently compromising the area, making it more susceptible to infections and irritants.^7^ Other symptoms include lichenification together with crusting, edema, oozing, erythema, or erosions, and in severe instances, hair may be sparse or absent.^7^ Symptomatic control in vulvar dermatoses is currently gained by using potent and very potent topical corticosteroids, such as betamethasone valerate or clobetasol propionate ointment, which are considered first-line treatments for LS and LSC.^8^ Although they show effectiveness in reducing symptoms to a level that is tolerable, side effects are to be taken into consideration.^9^

GSM, previously known as vaginal atrophy,^10^ is the most common vulvovaginal condition in postmenopausal women.^11^ The disease is triggered by a shortage in estrogen, which causes cell substance reduction, as well as epithelial and mucosal thinning due to loss of elastin, collagen, glandular secretions and blood vessel supply, thereby reducing vaginal secretions and vascularity, as well as inducing changes to the vaginal microflora.^12^ The symptomatic consequences include vaginal dryness, itching, irritation, and dyspareunia. When the epithelium becomes inflamed, the urinary tract may be affected by incontinence and recurrent infection.^13,14^ Due to the hormonal etiology, the usual course of treatment for GSM is hormone therapy (HT), commonly estrogen therapy (ET).^12^

Long-term treatment with corticosteroids and ET can potentially cause severe side effects if absorbed systematically such as atrophy, weight gain, breast tenderness, vaginal bleeding, and nausea.^12^ The possibility of side effects, paired with various contraindications for ET use, likely accounts for the observed reluctance toward standard treatments.^12,14^ However, available treatment alternatives are often associated with short- or long-term side effects themselves or show poor clinical results in improving the condition and relieving symptoms.^14,15^

Wound dressings formulated as film-forming silicone gels are well known to be safe and effective in long-term treatment of use on open wounds and de-epithelialized skin.^16,17^ Anecdotal evidence suggests that these products might also improve signs and symptoms associated with vulvovaginal conditions, due to their semi-occlusive characteristics that increase skin hydration, reduce the skin’s inflammatory response, and regulate fibroblast production.^18,19^ The studied topical gel is applied from a tube, inert and no adverse reactions have been reported to date.^19^ Nevertheless, the acceptability and potential benefits of this medical device as a symptomatic treatment option for vulvovaginal conditions has not yet been studied. Therefore, this study aimed to investigate the effects of this newly available topical gel in the management of signs and symptoms associated with vulvovaginal skin conditions in female patients.

## Methods

The present study was designed as a single-arm, open-label intervention study to investigate changes in clinical signs and symptoms among women with vulvovaginal skin conditions upon internal and/or external application of a newly available topical silicone gel (StrataMGT, Stratpharma AG, Switzerland), composed of polydimethylsiloxanes, siloxanes, and alkylmethyl silicones. All study-related procedures were performed in accordance with the Declaration of Helsinki and followed the ICH E6 (R2) Good clinical practice guidelines. Ethics approval was obtained before commencement of the study by the Allendale Investigational Review Board and the study was published in ClinicalTrials.gov with number NCT05396261.

Fifty-five women with diagnosed LS, LSC, or GSM from the private consulting suites “The Pelvic Medicine Center” within St. Andrews War Memorial Hospital were enrolled in this study. Eligible participants were women diagnosed with LS, LSC, or GSM, who currently had ongoing symptoms as diagnosed by themselves or the referring doctor for more than 3 years that were not being controlled by current treatment and who were able to provide informed consent. Patients with LS and LSC were mostly diagnosed clinically; biopsy was performed if there was any doubt in diagnosis.

Concomitant steroidal or HT was not an exclusion criterion, as the gel is primarily used as adjunct treatment that aims to improve persistent symptoms. A screening question was used to determine whether patients exhibited uncontrolled symptoms before study enrolment. Patients were considered to have uncontrolled symptoms if they had utilized betamethasone but discontinued due to the absence of desired symptom reduction or if they had intermittently used the medication without achieving symptom control. Detailed results of this screening process are presented in Table 1. Any concomitant medication was noted in the case report form. Participants were excluded if they were unable to apply the topical device or if they had an allergy or intolerance to any of the ingredients/excipients of the study device. Patients with a history of Candida before study enrolment were physically examined to ensure that they did not present a yeast or bacterial infection at the start of the study. If there was any doubt as to whether an infection may be present, the area was swabbed and sent for microbial assessment. New cases of Candida were monitored through adverse events reporting at every assessment visit for every patient.

**Table 1.** Patient Demographics and Diagnostic Information

	Lichen sclerosus	Lichen simplex	Genitourinary syndrome of menopause	All patients
Age (mean)	55.41	25	66.41	56.75
Diagnosis				
*n*	41	2	9	52
Mean age of diagnosis (years)	7.7	3.0	7.4	6.0
Chronic/Acute	38/3	2/0	9/0	49/3
Uncontrolled/Controlled	37/4	1/1	9/0	47/5
Secondary Diagnosis				
*n*	14	0	1	15
Genitourinary Syndrome of Menopause	13	0	0	13
Candida	1	0	0	1
Vulvodynia	0	0	1	1
Concomitant Medication^a^				
* n*	31	1	7	39
Corticosteroids	8	0	0	8
Hormones	26	1	7	33
Antifungals	2	0	0	2
Moisturizers	4	0	1	5

^a^ Row totals in all patients do not sum up to the total of 39 patients using concomitant medication, due to 9 patients (8 LS, 1 GSM) using multiple medications.

Upon enrolment, patients were asked to apply the study product to the genital area, minimum two times per day, including after each washing or urination. During each application, 3–5 drops of gel were placed on a fingertip and evenly distributed internally and/or externally to the affected area.

The study was separated into 2 timeline groups. All patients who provided written consent entered the first group, which aimed to assess the study product as a new treatment method in a short-term timeline during 3 visits coinciding with the primary endpoint of the study. Study visits were scheduled at 1.5 months (V1), 3 months (V2), and 6 months (V3) after the start of treatment. Participants were invited to crossover to the long-term study group to continue assessing the gel during four subsequent visits (V4–V7; Table 2). For analysis, patients were further divided into subgroups according to their treating diagnosis.

**Table 2.** Timelines of Study Visits in Days from Treatment Start

Treatment period	Mean (days)	SD^a^ (days)
Short-Term *(n = 52)*		
Baseline	0	0
Visit 1 (V1)	44	20
Visit 2 (V2)	113	45
Visit 3 (V3)	195	71
Long-Term *(n = 30)*		
Visit 4 (V4)	411	249
Visit 5 (V5)	502	63
Visit 6 (V6)	586	60
Visit 7 (V7)	665	35

^a^ SD = standard deviation.

Patients were assessed during disease-specific standard visits. Due to a lack of validated scales, a 10-point Likert scale ranging from 0 = “normal” to 10 = “worst possible” was specifically designed for this study and used by the investigator to rate the clinical signs and the visual severity of the condition, as well as to capture patient-reported outcome measurements (PROM) (see Supplementary Fig. S1).

One single investigator was performing the study, who conducted outcome measurements and assessed the degree of pathology visually by diagnosing the severity of the clitoral hood, urethral area, vaginal vault, labia majora and minora, fourchette, perineum, perianal skin, extragenital site, and inguinal folds according to the physician’s clinical experience. Clinical signs were also captured at every visit, including dryness, tissue thinning, erosions/ulcers/fissures, erythema, adhesion/scarring, contact bleeding, blood blisters, labial fusion, grayish film, labial resorption, white lacy streaks, and others. PROM included the symptomatic assessment of pruritus, tenderness, swelling, dryness, burning of the skin, dyspareunia, stinging with urination, irritation with clothes, and pain during defecating. Patient adherence to the treatment was documented retrospectively at every visit. Full compliance (100% adherence) with the treatment regimen was based on the assumption of a twice-daily application for 7 days per week.

The number of adverse events that occurred during the short- and long-term trial period was recorded at all visits.

According to the statistical analysis plan, symptomatic improvement for both short- and long-term patients was analyzed using nonparametric Friedman test using the Bonferroni correction for multi-comparison in SPSS version 24. Both severity of pathology and clinical signs were pooled for analysis, *i.e.,* for each time point the average severity over all pathological regions and the average severity over all clinical signs were calculated per patient. Average severity was then compared over time, although only data corresponding to the short-term group were available for scrutiny applying the same nonparametric test. Finally, patients were split according to their diagnosis type for subgroup analysis, which was performed as described above.

## Results

In total, 52 out of 55 women with a mean (SD) age of 56.8 (±15.1) years applied the gel as recommended and were included for analysis. Two patients withdrew voluntarily after the first study visit, as they preferred the previous product they had been using and 1 patient was lost to follow-up. All patients completed baseline assessment as well as three follow-up visits (short-term). Thirty patients continued the study and completed all four additional follow-up visits (long-term) as described in Table 2.

Forty-one out of 52 women had a primary diagnosis of LS (78.9%), 2 had LSC (3.8%), and 9 had GSM (17.3%) (Table 1). Of these patients, 49 (94.2%) suffered from a chronic disease and 3 cases (5.8%) were acute. Forty-seven out of 52 patients (90.4%) suffered from uncontrolled disease (Table 1).

**FIG. 1.**
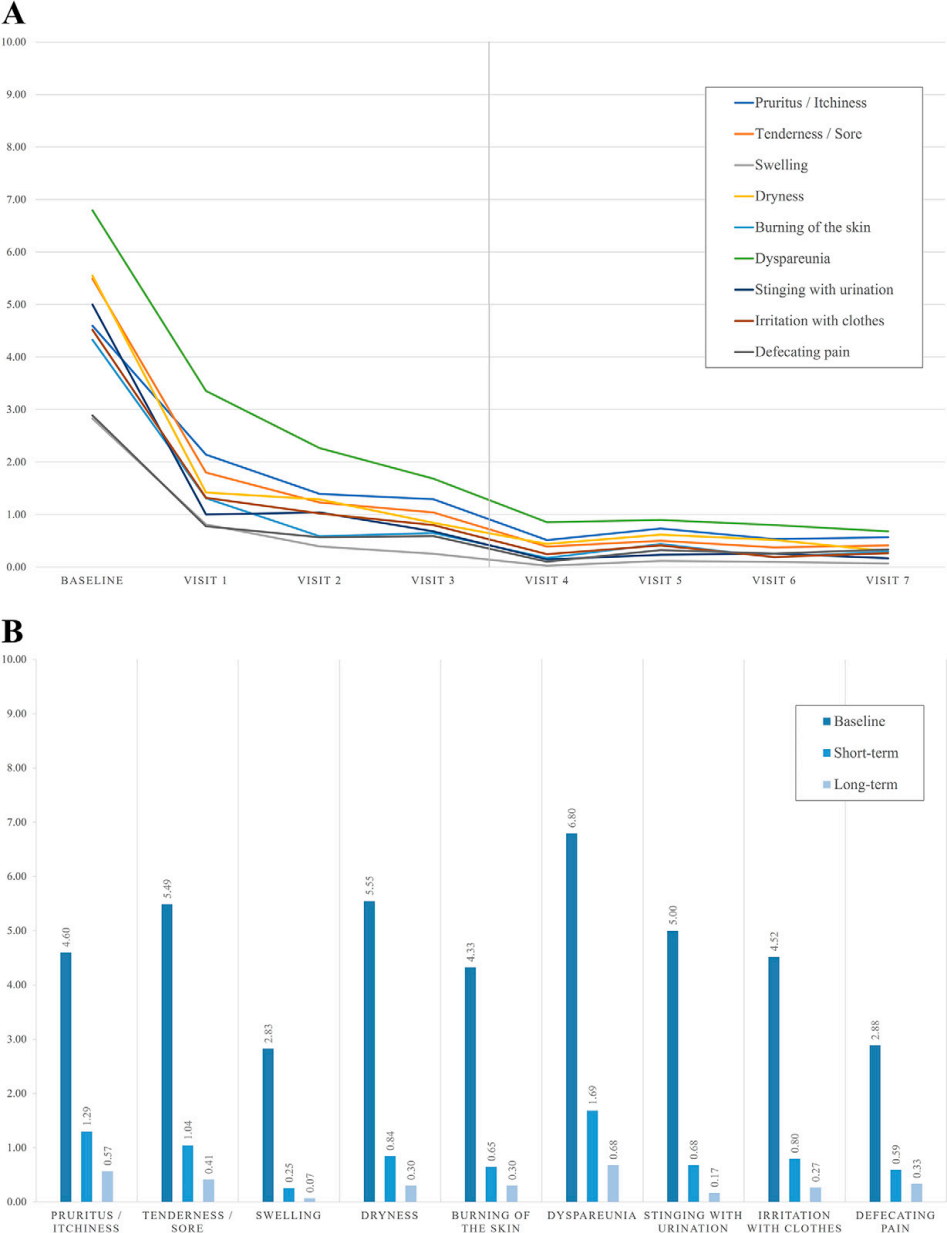
Symptoms over time for all patients. **(A)** Symptom severity was rated by the patient during each visit and visualized over time. The vertical gray line indicates the separation between short-term (*Baseline* to *Visit 3*) and long-term (*Visit 4* to *Visit 7*) assessments. **(B)** Symptomatic improvement was analyzed using the nonparametric Friedman test. All symptoms showed significant improvement at the short-term assessment (up to 6 months) and the favorable results were maintained until the end of the long-term assessment (up to 2 years).

All PROM significantly decreased during the short-term treatment (*p* < 0.001) (Table 3; Fig. 1). Thereby, the most significant improvement was seen for all patients from baseline to V1 (*p* < 0.001; Fig. 1a), where symptoms improved up to a 100% resolution for some patients. At the end of the short-term treatment, 17 patients (32.7%) had complete remission of symptoms. Overall, the strongest reduction in symptom severity score was observed in dyspareunia, where the baseline score of 6.80 decreased to 1.69 (*p* < 0.001) after 6 months of treatment, which equals in a reduction of 75.18% in average symptom severity (Fig. 1b). The clinical control of all other symptoms was maintained for up to 2 years of treatment, with the long-term Friedman analysis showing no more statistical changes in the dataset over the entire prospective period (Table 3).

**Table 3.** Patient-Reported Symptoms for Short- and Long-Term Groups, Analyzed Using the Nonparametric Friedman Test

	Short-term						Long-term					
	n	Baseline Mean (SD^a^)	Visit 1 Mean (SD^a^)	Visit 2 Mean (SD^a^)	Visit 3 Mean (SD^a^)	Significance (Baseline—Visit 3)	n	Visit 4 Mean (SD^a^)	Visit 5 Mean (SD^a^)	Visit 6 Mean (SD^a^)	Visit 7 Mean (SD^a^)	Significance (Visit 3 —Visit 7)
Pruritus	48	4.60 (3.73)	2.14 (2.66)	1.39 (2.20)	1.29 (2.32)	<0.001^*^	28	0.51 (0.98)	0.74 (1.29)	0.53 (1.08)	0.57 (1.01)	0.957
Tenderness	49	5.49 (3.19)	1.80 (2.51)	1.23 (2.09)	1.04 (2.40)	<0.001^*^	27	0.39 (0.86)	0.50 (1.52)	0.38 (0.79)	0.41 (1.15)	0.884
Swelling	47	2.83 (3.61)	0.81 (1.96)	0.39 (1.22)	0.25 (1.20)	<0.001^*^	27	0.02 (0.16)	0.12 (0.69)	0.10 (0.40)	0.07 (0.37)	0.985
Dryness	49	5.55 (3.55)	1.42 (2.54)	1.29 (2.45)	0.84 (2.09)	<0.001^*^	27	0.44 (1.27)	0.62 (1.72)	0.52 (1.21)	0.30 (1.02)	0.170
Burning of the skin	47	4.33 (3.51)	1.31 (2.50)	0.59 (1.77)	0.65 (1.71)	<0.001^*^	28	0.17 (0.59)	0.44 (1.35)	0.19 (0.47)	0.30 (0.84)	0.696
Dyspareunia	26	6.80 (3.99)	3.35 (3.68)	2.26 (3.35)	1.69 (2.81)	<0.001^*^	19	0.85 (1.91)	0.90 (2.23)	0.80 (2.18)	0.68 (2.19)	0.004^*^
Stinging with urination	48	5.00 (3.86)	1.00 (2.25)	1.04 (2.22)	0.68 (1.74)	<0.001^*^	27	0.15 (0.53)	0.24 (0.55)	0.26 (0.89)	0.17 (0.59)	0.368
Irritation with clothes	48	4.52 (3.70)	1.32 (2.29)	1.02 (1.95)	0.80 (1.54)	<0.001^*^	27	0.24 (0.73)	0.41 (1.48)	0.19 (0.60)	0.27 (0.87)	0.379
Defecating pain	47	2.88 (3.60)	0.78 (2.15)	0.57 (1.63)	0.59 (1.42)	<0.001^*^	26	0.10 (0.38)	0.32 (1.04)	0.26 (0.77)	0.33 (0.88)	0.555

^a^ SD: standard deviation.

^*^ : significant as per α = 0.05.

At the end of the short-term trial, the pooled clinical signs and pooled visual severity of pathology were significantly improved compared with baseline, on average from 4.86 to 1.64 (*p* < 0.001) and from 5.30 to 2.34 (*p* = 0.004). In detail, 7 of 13 clinical signs and 7 of the 10 assessed locations in visual pathology showed statistically significant improvement (see Table 4).

**Table 4.** Clinical Signs and Visual Severity of Pathology of the Short-Term Trial, Analyzed Using the Nonparametric Friedman Test

Outcome measure	n	Baseline	Visit 1	Visit 2	Visit 3	Significance (Baseline—Visit 3)
Visual Severity of Pathology						
Clitoral Hood	10	6.10	3.12	2.32	2.06	0.001^*^
Urethra Area	10	5.10	2.48	2.00	1.69	<0.001^*^
Vaginal Vault	4	3.82	1.25	2.00	1.67	0.392
Labia Majora	14	5.30	2.32	2.29	2.36	<0.001^*^
Labia Minora	16	6.40	3.48	2.38	2.88	<0.001^*^
Fourchette	14	5.63	2.67	2.19	2.35	<0.001^*^
Perineum	14	5.34	2.50	2.00	2.71	<0.001^*^
Perianal SKIN	8	4.00	2.29	0.91	2.45	0.008^*^
Extragenital SITE	3	0.86	0.00	0.00	0.00	1.000
Inguinal FOLDS	3	1.00	0.00	0.00	0.00	1.000
Clinical Signs						
Dryness	15	6.34	1.93	0.83	0.43	<0.001^*^
Tissue Thinning	18	6.25	3.38	2.15	1.83	<0.001^*^
Erosions/Ulcers	10	0.65	0.59	0.63	0.94	0.392
Fissures	15	2.74	0.63	0.12	0.23	0.001^*^
Erythema	19	3.79	1.75	1.08	1.13	<0.001^*^
Adhesion/ Scarring	10	2.14	0.73	0.18	0.40	0.392
Contact Bleeding	5	0.67	0.38	0.33	0.00	1.000
Blood Blisters	5	1.55	1.00	0.22	0.11	1.000
Labial Fusion	11	5.63	4.19	3.33	2.79	0.001^*^
Grayish Film	4	4.50	2.40	2.40	3.60	0.430
Labial Resorption	12	6.24	5.19	3.78	2.35	<0.001^*^
White Lacy Streaks	3	4.57	2.80	1.75	1.57	0.204
Other	8	7.14	3.58	2.55	1.56	<0.001^*^

^*^ : significant as per α = 0.05.

While the same improvement pattern is visible in the LS patient subgroup, the symptomatic improvement over time in patients diagnosed with GSM is significant using the Friedman Test, but not after the Bonferroni correction for multiple testing. The same statistical tests could not be performed on the LSC subgroup due to the too small sample size (*n* = 2; Table 1).

Verbally reported patient compliance with the newly studied product was high (from baseline to V7; Table 5). No product-related adverse events occurred throughout the study.

**Table 5.** Patient Compliance with Treatment

Treatment period	n	Treatment compliance
Baseline–Visit 1	50	97.0%
Visit 1–Visit 2	51	94.9%
Visit 2–Visit 3	46	88.4%
Visit 3–Visit 4	35	86.4%
Visit 4–Visit 5	36	95.9%
Visit 5–Visit 6	28	94.6%
Visit 6–Visit 7	22	93.5%

## Discussion

PROM, clinical signs, and visual severity of pathology were overall improved by the studied gel across the investigated vulvovaginal skin conditions. The strongest improvement was observed during the first 1.5 months of treatment with progressive improvement thereafter until the end of the short-term assessment. With continuous application of the study product, the favorable results were maintained for nearly 2 years. Treatment adherence was exceptionally high, and no adverse events related to the study product occurred.

These findings agree with previous literature showing reduced erythema and inflammation, as well as an accelerated wound healing effect of similar topical gels in acute and chronic wounds by creating an optimal healing environment.^17,18^ This type of wound healing environment enhances angiogenesis, stimulates keratinocyte migration, reduces infection rates, allows for postinjury growth factors to remain intact, and does not disturb the superficial healing.^18^ For example, in a series of clinically challenging cases published by^18^ on nonhealing wounds, a semipermeable film-forming gel device was shown to decrease the postinflammatory burning sensation in all patients, while also reducing erythema and superficial skin temperature. Other studies found that it lightly adheres to the healing tissue, creating a protective barrier film that reduces mechanical friction, thereby preserving the healing epidermis from external disrupting influences.^16,20^ Fully protecting the affected areas not only reduces potential infectious complications and allows for a controlled healing environment but also provides a soothing effect that relieves symptoms such as pruritus and pain related to dryness, which in turn contributes to positive patient outcomes and compliance.^16,17,20^

The studied gel is gas-permeable, semi-occlusive and bacteriostatic; properties that are believed to be beneficial to wound healing.^18,21^ It is also predicted to improve clinical signs and severity of pathology, primarily experienced with LS and LSC, by normalizing the production of collagen through the regulation of inflammatory growth factors—mainly transforming growth factor-β and interleukins. *In vitro* studies convey that through hydration, among other variables, these devices can directly regulate inflammatory growth factors involved in fibrosis and acute wound healing.^20^ Subsequently, the gel has promising potential to improve the underlying conditions and reduce the risk of skin thickening and fibrosis. The improvement of clinical signs and visual severity of pathology found in this study potentially provides early evidence to support these claims, which need to be studied further.

Patient-centered surveys investigating vulvovaginal symptoms on postmenopausal women have shown that the majority of patients (40%) rate their sexual life as the most negatively affected outcome influencing their sexual interest or libido (59%).^22,23^ Apart from physiological hormonal changes, the reduction in libido in postmenopausal women (87%) is linked with dyspareunia,^24^ which correlates with those patients also reporting dryness.^25^ Lubrication is the most common treatment, although dissatisfaction with this approach is also common, consequently engaging in potentially painful intercourse despite the vulvovaginal symptoms.^26^

When consulting a medical professional, the prescribed first-line treatments such as corticosteroids and ET typically present with a higher risk of side effects and are contraindicated in various other postmenopausal conditions.^12^ Second-line treatment options such as topical calcineurin inhibitors have demonstrated good efficacy,^12^ however, they are also associated with substantial side effects and have been reported to eventually increase the risk of vulvar malignancy.^27^ In contrast, the studied device achieved a statistically significant reduction in dyspareunia of more than 75%, without causing any side effects. When compared with the placebo effects that were observed in previous clinical studies which range between 40%–60%, this result may highlight the potential clinical significance of the outcome.^28^

In addition to dyspareunia, the studied product statistically improved all other patient-reported outcomes, including burning sensation, itchiness and dryness, for a sustained time in both short- and long-term groups, while remaining well tolerated and with no gel-related adverse events reported. This favorable safety profile is likely based on the high biocompatibility rating of this technology, which, in addition, does not actively influence the pH value upon application of the skin or mucosal tissue.^19^

The 1-year treatment adherence in this study was over 90%, contrasting with the risk of compliance reported with the use of estrogen or steroid creams.^29^ Common pharmaceutical advice provided for topical steroids is to use them sparingly due to their associated side effects. This risk-conscious advice, which is also often reported in the media, may lead to patients using the product under the therapeutic threshold, with subsequent negative symptom control.^30^ The studied device, however, shows minimal complication risk and is easy to use, thus improving treatment compliance.^18^ Meanwhile, the regular scheduled follow-up visits in this clinical trial together with the patients’ awareness of a potential symptomatic improvement in their chronic condition may have maximized the adherence to the investigational product. Moreover, the high number of times that the patients applied the gel per day is noteworthy, which exceeded the threshold required for full treatment adherence. Further research should focus on long-lasting formulations that are not only effective but require less frequent applications to minimize disruption to patients’ daily activities.

To our knowledge, this is the first study to investigate the potential benefits of this gel device in the symptomatic management of vulvovaginal skin conditions, such as LS, LSC, and GSM. The strength of this study is in its detailed and long-term assessment of both patient- and investigator-rated outcomes, while the main limitation is the absence of a control group. In addition, bias might have been introduced as the investigator also served as outcome assessor and thus was not blinded to the treatment. Hence, double-blinded randomized controlled trials that further investigate the findings of this feasibility study are required to compare the efficacy of the investigational product to standard of care. Other limitations of this study include the use of non-validated scales and the lack of recruitment into LSC and GSM study subgroups, which rendered statistical subgroup analysis difficult due to the small sample size. Therefore, future studies should account for a bigger sample size and equal-sized patient samples across the different indications.

Additionally, future research should include assessments of changes in both the internal and external microbiome of the treated area before and after product application. Understanding these microbiome shifts is crucial as they may impact patient outcomes. It is also important to note that “external vulvar tissue” and “internal vaginal tissue” have distinct compositions and microbiomes, highlighting the need to consider these differences in study designs.

## Conclusion

Following its successful use in wounds and compromised skin conditions, this feasibility study provides initial evidence for the use of this medical device for the symptomatic management of skin conditions in the vulvovaginal area. The favorable results suggest that it could be a promising, safer, and permanent adjunct treatment option for most women suffering from various highly symptomatic vulvovaginal conditions. Women with a personal history of previous estrogen-dependent cancer, or those who have been contraindicated to use ET or steroids, as well as patients refraining from these standard treatment options might particularly benefit from this newly emerged treatment.

## Supplementary Material

Supplementary Figure S1

## Data Availability

The data that support the findings of this study are available from the corresponding author, upon reasonable request.

## Abbreviations Used

ET: Estrogen Therapy
GSM: Genitourinary Syndrome of Menopause
HT: Hormone Therapy
LS: Lichen Sclerosus
LSC: Lichen Simplex Chronicus
PROM: Patient-Reported Outcome Measures
SD: Standard Deviation
V: Visit
