# Supplementary figures and images for: A Feasibility Study Investigating a Topical Preparation as Novel Adjunct Treatment for the Symptomatic Management of Vulvovaginal Skin Conditions
Source: Womens Health Rep (New Rochelle). 2024 May 17;5(1):444–52. doi: 10.1089/whr.2024.0026 (PMC11257114; doi:10.1089/whr.2024.0026)

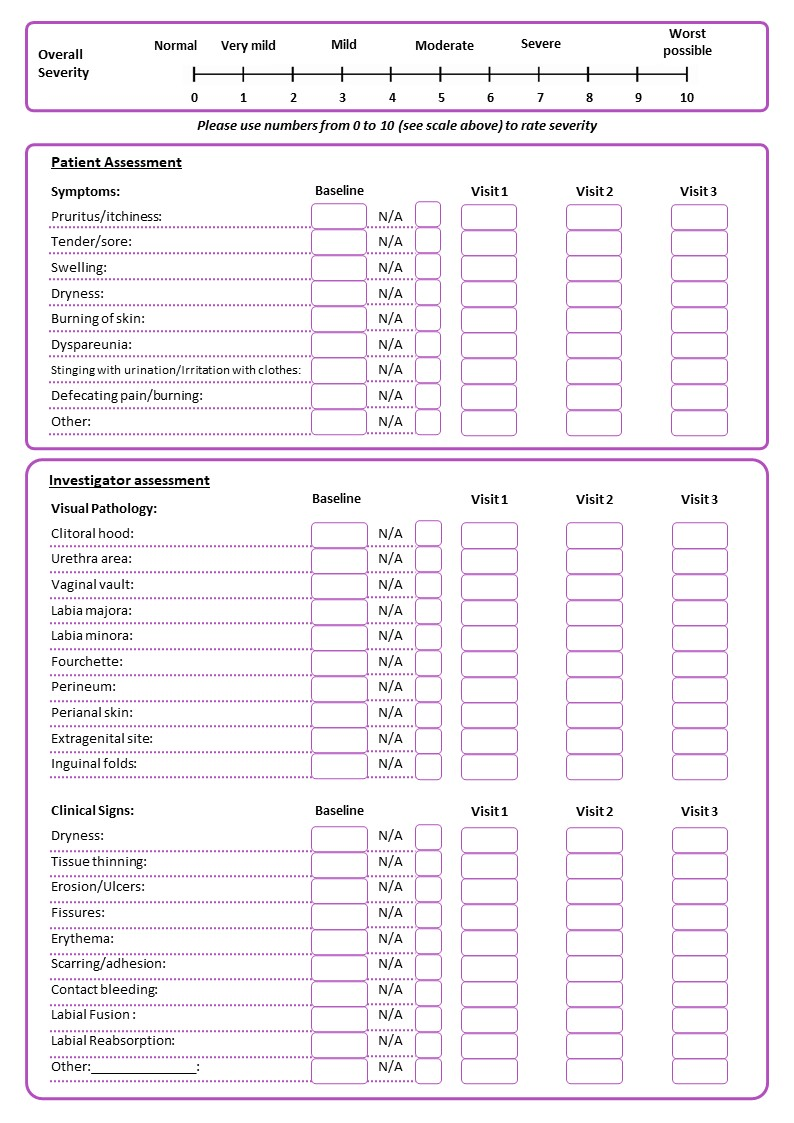

Supplement: Supplementary Figure S1 [file whr.2024.0026_supplfig1.tiff]
